# Texturized Vegetable Protein as a Source of Protein Fortification of Wheat Buns

**DOI:** 10.3390/foods11223647

**Published:** 2022-11-15

**Authors:** Susanne Bølling Laugesen, Sandra Lenz Dethlefsen, Iben Lykke Petersen, Margit Dall Aaslyng

**Affiliations:** 1 Nutrition and Health, Centre for Nutrition, Rehabilitation and Midwifery, University College Absalon, Sdr. Stationsvej 30, DK-4200 Slagelse, Denmark; 2 Department of Food Science, University of Copenhagen, Rolighedsvej 26, DK-1958 Frederiksberg, Denmark

**Keywords:** pea protein, faba bean protein, hemp protein, quinoa protein, protein extrudates, texturized vegetable protein, older adults, bread, protein fortification

## Abstract

Increasing interest in plant-based proteins is particularly relevant in the food service sector. For specific groups, e.g., older adults, it may be challenging to ensure the consumption of protein of sufficient quality. One way of doing this could be through the fortification of a staple food such as bread. This study examined wheat buns, in which 0%, 20%, 35% and 50% of the flour was replaced with three different milled texturized vegetable proteins (TVP) of different plant protein combinations. Sensory and baking qualities were evaluated through sensory profiling and measurements of rising ability, baking loss, protein content and colour. An expert assessment and a robustness test were conducted to evaluate potential use in the food service sector. By substituting 35% of the wheat flour with milled TVP, it was possible to increase the protein content of the buns by 83% (up to 25% of DM) and still maintain an acceptable quality. The different TVPs showed that pea and faba bean or pea, faba bean and quinoa were more suitable in bread fortification than pea, faba bean and hemp. The study demonstrates the potential for producing quality bread for people who need a high protein intake in all their meals.

## 1. Introduction

Interest is growing among both individuals and the public in general in reducing the consumption of food of animal origin and instead increasing that of plant-based food. In Denmark, new dietary recommendations advise eating as little as 350 g of meat per week and increasing the intake of pulses [1]. A plant-based diet has several positive health effects, and a vegetarian or vegan diet often scores higher in overall health quality (e.g., measured by the healthy eating index, HEI) compared with a diet including meat [2,3,4,5,6]. However, in a plant-based diet, particular emphasis must be placed on protein availability. The protein quality of plant proteins is generally lower than that of animal proteins, since the amino acid composition is incomplete and digestibility is reduced, thus lowering the bioavailability [7]. At the same time, the protein intake is often lower in plant-based diets compared with animal-based diets even though it is still within the recommended level [6].

Plant proteins have less anabolic effect compared with animal proteins [8]. In older adults in particular, protein is important for preventing loss of muscle mass, so-called sarcopenia [9]. Older adults therefore require a high content of easily digestible protein with a balanced amino acid composition in their diet, preferably in all the main meals of the day [9]. When aiming to reduce animal protein in the diet, also in the public food service sector, this must be taken into account during meal planning to ensure sufficient protein of high quality. One way of improving the quality of a meal is to combine different protein sources with supplementary amino acid composition. Furthermore, technological processing can increase the bioavailability of the protein by enhancing digestibility and reducing the anti-nutritional components [8].

Extrusion is a technological low processing technique in which the protein is transformed through several mechanical steps into a snack-like or meat analogue-like product with increased protein digestibility and increased water-binding capacity [10]. Improvement of the native protein digestibility is thus achieved through the denaturation of the proteins, resulting in exposing the amino acids more cleavage sites making them available for digestive enzymes, and by reducing or removing anti-nutritional components in the native plant material [11,12]. It is possible to produce a higher nutritional quality by combining different protein sources before extrusion with protein from pulses containing high amounts of lysine [13,14] and protein from cereals, pseudocereals or oilseeds with high amounts of the sulphur-containing amino acids methionine and cysteine [7]. This makes texturized vegetable proteins (TVPs) superior to the native proteins. Texturized vegetable protein (TVP) has the potential to be used not only for plant-based meat analogues but also as protein-fortifying ingredients in different food items.

In older adults, breakfast has been identified as a meal in which the protein content is generally low, while the carbohydrate content is high [9]. Using milled TVP to increase the nutritional value of bread for breakfast is therefore of particular interest for this group of people. However, since the baking quality of bread depends on the gluten content [15], this can be expected to decrease when part of the flour is replaced by milled texturized vegetable protein, which does not contain gluten.

The aim of the current study was therefore to investigate the effect of replacing part of the wheat flour with milled TVP in buns on the quality depending on the amount of TVP (0%, 20%, 35% and 50% of the flour). Both the sensory quality and the baking quality were assessed. Three different TVPs were used to evaluate the effect of different plant protein combinations. In order to evaluate the robustness of the buns for use in the food service sector, both the effect of storage method and defrosting method were investigated.

## 2. Materials and Methods

### 2.1. Ingredients

Three TVPs based on different combinations of raw ingredients were used: TVP1 (10% faba bean, 89% pea, 1% salt), TVP2 (49% faba bean, 40% pea, 10% quinoa, 1% salt) and TVP3 (10% faba bean, 79% pea, 10% hemp, 1% salt) (Organic Plant Protein A/S, Hedensted, Denmark). The protein sources were combined to obtain a comparable protein content and a full amino acid score. The extrusion was performed at Organic Plant Protein A/S using a commercial twin screw extruder with a die plate of 2.5 mm × 22 mm, a 16 mm diffuser, two knives and 2100 RPM, giving the extrudates a specific gravity of 170–180 g/L for TVP1 and TVP2 and 180–200 g/L for TVP3.

Before use, all extrudates were ground into flour using a Magimix 11,610 food blender (Magimix, Vincennes, Ile-de-France, France) at maximum speed for two minutes, after which the flour was sieved to ensure that all material was milled to a particle size that could pass through a medium sieve with mesh size 12 (approximate hole size 1.7 mm).

### 2.2. Recipe and Bun Production

The recipes were developed in a pre-study with the aim of creating a vegan bun with focus on volume.

In total, ten different types of buns were prepared: a reference bun (REF) based on Manitoba wheat flour (Molino Grassi Spa, Italy) without the addition of TVP, and nine buns containing TVP1, TVP2 or TVP3 in concentrations of 20%, 35% and 50% of the flour, respectively. Manitoba wheat flour, which has a high glutenin and gliadin content, was chosen to optimise the baking quality. The recipe for the buns is described in Table 1.

First, oat milk, dry yeast and sugar were mixed using a TEDDY kneader (Varimixer A/S, Denmark) with a hook, followed by a ten-minute resting period. Next, aquafaba was added, and the mix was stirred for one to two minutes until a white foam appeared. TVP flour, Manitoba wheat flour and salt were mixed manually in a separate bowl. The dry ingredients were added to the wet mixture in the TEDDY kneader and mixed together, and then soft vegan butter was added. After all the ingredients had been mixed for ten minutes using the TEDDY kneader with a hook at the second-highest speed, the mixing bowl was covered with plastic wrap, and the dough was left to rise at room temperature for 45 min.

The dough was then transferred to a flour-covered table and divided into equal pieces, each weighing exactly 50 g, hand-rounded into buns and placed on a parchment-lined baking tray. The baking tray was covered in plastic wrap, and the buns were left to rise for 45 min. After rising, the buns were placed in the centre of a preheated electric oven (iCombi Classic, RATIONAL Scandinavia AB, Malmö, Sweden) and baked at 200 °C and 100% relative humidity (RH) for approximately ten minutes (REF), 12 min (20% TVP and 35% TVP) or 15 min (50% TVP) to a core temperature of 93–95 °C. After baking, the buns were left to cool on a rack for approximately 30 min. The same oven was used for baking of all the buns.

Three replicates of dough were prepared for the sensory profiling with a batch size of approximately 1 kg dough per bun type.

### 2.3. Baking Quality

Weight loss was calculated by weighing the dough before baking (exactly 50 g per bun) and approximately 30 min after baking.

The rising ability was assessed by measuring the diameter of the bun with a calliper at three random horizontal points on the bun to obtain an average diameter. The bun was then cut in half vertically, and the height was measured with the calliper at the top centre of the cut surface. An approximation of the apparent volume was calculated using Equation (1), where *V* is the volume, *h* the bun height in cm and *D* the bun diameter in cm. All of the above-mentioned measurements were collected in five replicates.

*V* = 1/3 *π h*^2^ (3/2 *D* − *h*) (1)

Colour was measured using a PCE-CSM 6 colorimeter (PCE Instruments UK Ltd., Hampshire, UK). The results were expressed in the CIELAB colour space, and calibration white was used as a standard. Colour determinations were performed for each dough on a cut surface at three points (triangulated), providing an average measurement, and in five replicates for both the crust and crumb of each bun, with the colour measured at five different points (in square formation and centre point) to provide an average measurement.

The protein content and dry matter were analysed in triplicate in one bun of each type. For protein content determination, milled samples were analysed using an Elementar vario MACRO cube CHNS analyser (Elementar Analysensysteme GmbH, Hanau, DE, USA) with helium as the carrier gas. The CHNS elemental analysis is based on the Dumas combustion method. The crude protein content was calculated from total nitrogen using a standard conversion factor of 6.25.

The dry matter (DM) content of the milled samples was determined using an HB43 Halogen Moisture Analyser (Mettler Toledo, Columbus, OH, USA). Drying was performed using a quartz heater at 105 °C until the variation of mass was less than ±0.001 g over a period of 30 s and the starting weight of the samples was 2.0−3.0 g.

### 2.4. Sensory Descriptive Analysis

A sensory panel consisting of six trained assessors (3 females/3 males, aged between 25 and 29 years) screened for sensory accuracy was used to obtain a sensory profile of all ten buns. All assessors had experience in sensory descriptive analysis of bread and consequently the panel discussion and training were compressed into two sessions of two hours per session. The assessors were trained using the samples, and descriptors were chosen based on suggestions from the panel leader and consensus among the assessors. Consensus was achieved through reference material (hazelnuts, wet cardboard, fresh yeast, hay, wheat flour, TVPs, rye flour and hemp flour). The performance of each individual assessor was evaluated relative to the performance of the entire panel to determine the sufficiency of training.

A modified quantitative descriptive analysis (QDA) was performed under normal lighting conditions in sensory boots at University College Absalon. All ten types of buns were evaluated with two replicates. All samples were coded with random three-digit numbers and served monadically in a balanced randomised order. Each assessor evaluated 27 descriptors (Table 2) on a 15 cm line scale with anchors at 1.5 cm (slightly) to 13.5 cm (much). Sparkling water and cucumber sticks were available for cleansing the palate.

### 2.5. Expert Evaluation

Twelve experts from the food industry and food service sector responsible for food for older adults evaluated four of the buns: the reference and TVP1-3 at 35%. Experts were selected as they are the gatekeepers of whether TVP-enriched bread will be offered in food service at all; furthermore, the end users of food service are older vulnerable adults, making a consumer test on this group ethically questionable.

The four particular buns were chosen to both investigate the effect of information about the plant proteins used, as well as serving buns with a maximized protein content that still had a relatively acceptable sensory quality. First, each expert was served the four buns in a randomised order and asked to evaluate how much he/she liked the bun on a modified 9-point hedonic scale [16] ranging from “Not at all” to “Like it a lot”. Next, the experts were given information about the amount and type of plant protein added to each bun and asked to evaluate the likelihood of using the bun professionally on a 9-point scale ranging from “Very unlikely” to “Very likely”.

### 2.6. Robustness of the Buns

In order to evaluate the robustness of the buns, a sensory evaluation was performed on buns that were freshly baked (D0), one day old (D1), frozen/thawed at room temperature (FT) and frozen/thawed in a microwave oven (FM). The reference bun and the buns containing 35% TVP2 (TVP2-35) were selected for this robustness test. Freshly baked buns (D0) were baked on the morning of evaluation and cooled on a rack for approximately 30 min, after which they were packed in a sealed freezer bag and stored at room temperature until sample preparation. The one-day old buns (D1) were baked the day before evaluation, cooled on a rack at room temperature for approximately 30 min, after which they were packed in a sealed freezer bag and stored at room temperature until sample preparation the next day. Two replicates of dough of the reference bun and the buns containing 35% TVP2 were prepared (for FT and FM) and frozen in a sealed freezer bag for approximately one week before testing. The buns thawed at room temperature (FT) were moved from the freezer to the kitchen table in the morning of evaluation to defrost at room temperature for at least four hours. The buns thawed in a microwave oven (FM) were taken directly from the freezer and thawed in a microwave oven (EPIQ digital microwave 700 W, 20 L capacity) on the defrost setting at medium intensity. The reference buns (REF) were microwaved for a total of 12 s (six seconds on each side). The buns with 35% TVP2 were microwaved for a total of 18 s (nine seconds on each side). To equalise the temperature throughout the bun, all buns were left to rest for approximately 15 min before serving. All buns were served at room temperature (23 °C ± 1 °C).

The evaluation was performed by a sensory panel consisting of six trained assessors (3 females/3 males, aged between 23 and 25 years) screened for sensory accuracy, two of whom had previously participated in the sensory analysis of all ten buns. As in the previous sensory descriptive analysis (Section 2.4), all assessors had experience in sensory descriptive analysis of bread and thus the panel was trained in two sessions of two hours per session. The assessors were trained using the samples, and descriptors were chosen based on suggestions from the panel leader and consensus among the assessors before conducting the final descriptive analysis (a modified QDA) under normal lighting conditions in sensory boots. All eight samples were evaluated in two replicates for 26 descriptors (Table 2).

### 2.7. Statistical Analysis

The statistical analysis was performed with the software programs PanelCheck v1.4.2, R v4.1.1 and RStudio v1.4.1717.

The sensory attributes of the increasing concentrations of TVP and the baking quality were initially analysed using the following model:

*Y_abc_* = *μ* + *C_a_* + *T_b_* + *CT_ab_* + *A_c_* + *ε_abc_* (2)

where *Y_abc_* is the (*abc*)th observation, *µ* is the general mean, *C_a_* and *T_b_* are the main effects of concentration and type of TVP, and *CT_ab_* is their interaction effect. These are all fixed effects. *A_c_* is the main effect of assessor, and *ε_abc_* is the random error. These are both random effects. When using this model, REF was not included in the investigation of interaction effects between concentration and type.

The following model was used to investigate the sample effects:

*Y_ab_* = *μ* + *S_a_* + *A_b_* + *SA_ab_* + *ε_ab_* (3)

where *Y_ab_* is the (*ab*)th observation, *µ* is the general mean, *S_a_* is the main effect of the sample, which is a fixed effect. *A_c_* is the main effect of assessor, *SA_ab_* is the interaction effect, and *ε_ab_* is the random error. These are all random effects.

## 3. Results

### 3.1. Baking Quality

Initially, it was observed that with increasing levels of TVPs in the dough, increasing amounts of oat milk was needed as the TVPs had a higher water uptake than the wheat flour. This meant that the amount of oat milk was different in the different recipes and that the water content of the dough could be expected to be different. However, this was necessary in optimization of the quality of the dough and the buns.

During baking, water evaporated from the buns. The baking loss was up to 10% of the weight (Table 3). A small but significant interaction was seen between the three types of TVPs and the concentration of TVP fortification (*p* = 0.02). No difference was found between the three concentrations of TVP2, while the baking loss was lower at 20% TVP compared with 35% and 50% of TVP1 and TVP3. Comparison with the reference bun showed that the baking loss was higher in the reference bun than in the buns with TVP, although the difference was only significant between the reference bun and the buns with TVP with the lowest baking loss.

All samples had a similar weight before baking, and the volume is therefore a reflection of the rising ability. All buns with TVP had a lower volume than the reference bun (*p* < 0.001) (Table 3). Furthermore, increasing amounts of TVP led to a decrease in the volume. There was a significant interaction between TVP and the concentration of TVP fortification (*p* < 0.001). TVP1 had a higher volume than TVP2 and TVP3 at 20% and 35%, while no differences in volume were detected between the three buns containing 50% TVPs. No differences were found between TVP2 and TVP3, independent of concentration.

The TVPs were analysed to confirm the comparable protein content. The protein contents analysed in the TVPs were: 51.2% (TVP1), 52.3% (TVP2) and 51.5% (TVP3), showing minor acceptable content differences. The protein analysis of the buns confirmed that increased addition of TVP led to a significant increase in bun protein content. No significant difference between TVP types was observed except at 35% addition, where TVP1-35 had a significantly lower protein content than TVP2-35 (*p* = 0.002) and TVP3-35 (*p* = 0.042). Substituting 20% Manitoba wheat flour with TVP increased the protein content by an average of 49% (from 13.7% of DM up to 20.4% of DM), while 35% TVP increased the protein content by an average of 87% (from 13.7% of DM up to 25.6% of DM). The addition of 50% TVP more than doubled the protein content, increasing it by 119% (from 13.7% of DM up to 30.0% of DM).

The colour of the dough was measured before baking, and the colour of the crust and crumb of the baked buns was measured (Table 3). Adding TVP to the dough gave a lower L*-value (darker) and higher a* (more red) and b* (more yellow) values, and the more TVP that was added, the larger the change. The biggest change was seen in the a*-value, which increased from 1.9 up to 10.6. The same pattern was seen in the crumb, and only minor differences were seen between the dough and the crumb, indicating that the colour of the crumb was directly affected by the colour of the dough. In contrast, the colour of the crust was only different from the REF in the L*-value, which was lower, while no differences were seen in the a* and b* values.

### 3.2. Sensory Descriptive Analysis

**Figure 1:**
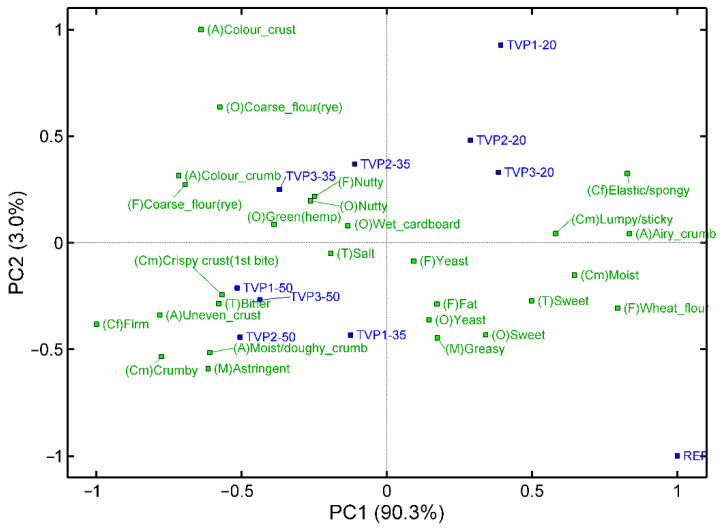
PCA biplot for the sensory profile of all buns. REF: Reference sample with 0% added TVP; TVP1: extrudate with 10% faba bean, 89% pea, 1% salt; TVP2: extrudate with 49% faba bean, 40% pea, 10% quinoa, 1% salt; TVP3: extrudate with 10% faba bean, 79% pea, 10% hemp, 1% salt; 20: 20% addition; 35: 35% addition; 50: 50% addition. A: appearance, O: odour, F: flavour, T: taste, Cf: consistency with fingers, Cm: consistency in mouth, and M: mouthfeel.

Initially, the three levels of TVP for all three TVPs were evaluated together with a reference bun without TVP. The full data table can be seen in the Supplementary Table S1. A principal component analysis (PCA) was performed to evaluate the overall variation in the sensory data (Figure 1). PC1 explained the main part of the variation (90.3%) and represented the increasing concentrations of TVP ranging from the reference (0%) to 50% TVP. The sensory profile shifted from an elastic/spongy, sticky sample with a wheat flour flavour towards a firm, dark, astringent and bitter bun. The differences between the three TVPs were smaller and were represented by a combination of PC1 and PC2. In Figure 1, it can be seen that at 20% TVP1 was evaluated as having a darker colour at the crust than TVP2 and TVP3, but the opposite was the case at 35%. At 35%, however, TVP1 seemed to be more astringent than the other two TVPs. The difference between TVP3 35% and 50% in PC2, which explains only 3% of the variation, indicating that, with the main part of the variation, these two samples did not differ. TVP3 therefore seems to be more vulnerable and provides a sensory quality that is more distinct from the reference already at 35% compared with TVP1 and TVP2.

**Figure 2:**
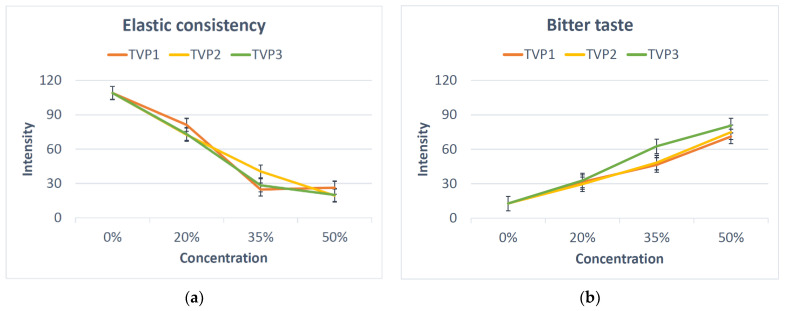
The intensity of the relevant sensory properties (**a**) elastic consistency and (**b**) bitter taste in relation to added type and concentration of plant protein. The results for the reference (REF) are indicated at 0%. TVP1: extrudate with 10% faba bean, 89% pea, 1% salt; TVP2: extrudate with 49% faba bean, 40% pea, 10% quinoa, 1% salt; TVP3: extrudate with 10% faba bean, 79% pea, 10% hemp, 1% salt. The standard error (SE) for the two repetitions is shown in the figure.

Even though most of the variation was between the concentrations, minor differences were also seen between the three TVPs (Supplementary Table S1). A change in attribute intensity with increasing concentrations can be seen when looking at the two selected attributes in Figure 2. At 35% substitution with TVP2, the bun had a slightly more elastic consistency than the other two buns with 35% TVP. Furthermore, the substitution with 35% TVP3 resulted in a more bitter taste than when TVP1 or TVP2 were used at the same level of concentration. Balancing the need for the highest possible protein concentration with an acceptable sensory quality, TVP2-35 was chosen for the robustness test and TVP1-3 at 35% for the expert evaluation.

### 3.3. Expert Evaluation

The results from the expert evaluation of the buns can be seen in Table 4. Without any information, all samples were rated as approximately equally liked, except for TVP3-35, which had a lower average liking rating (not significant). With information on protein content and the amount substituted, the assessment of the likelihood of professional use revealed a shift between the samples, with the reference sample now being, on average, the least likely to be used (not significant).

### 3.4. Robustness of the Buns

**Figure 3:**
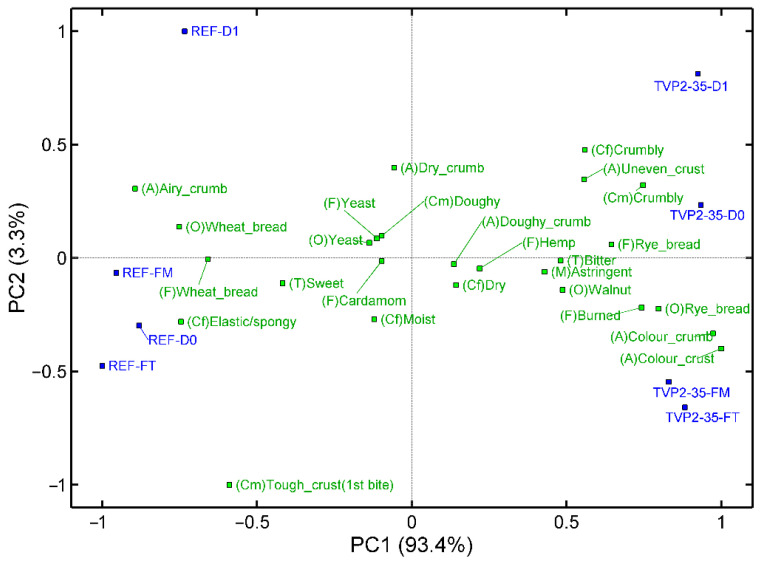
PCA biplot for the sensory profile of robustness of the selected buns. The reference (REF) had 0% added TVP; TVP1: extrudate with 10% faba bean, 89% pea, 1% salt; TVP2: extrudate with 49% faba bean, 40% pea, 10% quinoa, 1% salt; TVP3: Extrudate with 10% faba bean, 79% pea, 10% hemp, 1% salt; 20: 20% addition; 35: 35% addition; 50: 50% addition. A: appearance, O: odour, F: flavour, T: taste, Cf: consistency with fingers, Cm: consistency in mouth, and M: mouthfeel. D0: freshly baked, D1: one day old, FT: frozen and thawed at room temperature, FM: frozen and thawed in a microwave oven.

The robustness of the reference bun and TVP2-35 was evaluated by comparing buns that were freshly baked, buns after one day of storage, buns that were frozen for one week and thawed at room temperature and buns that were frozen for one week and thawed in a microwave oven. The full data table can be seen in the Supplementary Table S2. A PCA shows the overall variation in the sensory data (Figure 3).

Most of the variation in the data is explained by PC1 (93.4%) and relates to the differences between the reference buns and the TVP2-35 buns. The sensory profile of the reference buns is: airy crumb appearance, wheat odour and flavour, sweet taste, elastic/spongy texture and a tough crust at first bite. However, the TVP2-35 buns are described as: uneven crust, darker crust and crumb, rye bread odour and flavour, burnt odour and crumbly texture. PC2 explains 3.3% of the variation in the data and appears to be related to the effect of treatment. Buns stored for one day are located in the upper part of the plot and are described as having a crumbly texture. The difference between D0 and D1 is larger in the reference than in the TVP2-35. The buns frozen and thawed at room temperature are located in the lower part of the plot, with the reference being in close proximity to freshly baked and microwaved compared to TVP2-35, which is only in close proximity to microwaved. These buns are mainly described as having a tough crust at first bite and an elastic/spongy consistency.

**Figure 4:**
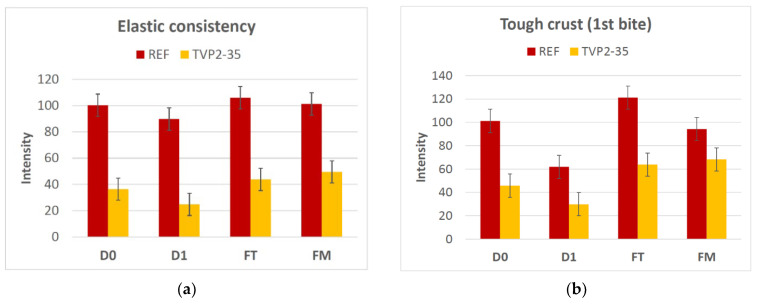
The intensity of the relevant sensory properties (**a**) elastic consistency and (**b**) tough crust at 1st bite in relation to robustness of the buns. The standard error (SE) for the two repetitions is shown in the figures. The reference bun (REF) contains 0% extrudate, and TVP2 contains 35% extrudate made from 49% faba bean, 40% pea, 10% quinoa, 1% salt. D0: freshly baked, D1: one day old, FT: frozen and thawed at room temperature, FM: frozen and thawed in a microwave oven.

On closer inspection of these particular attributes, it can be seen that one day of storage reduces the intensity of both attributes compared to freshly baked buns, whereas freezing and thawing either have no impact on, or even increase, the intensity (Figure 4).

## 4. Discussion

Increasing the consumption of protein of sufficient quality in all meals is a challenge, especially in older adults. For many older adults, breakfast is a carbohydrate-rich meal [9], and there is an untapped potential for increasing the intake of protein in this meal by increasing the protein content of the bread that is served [8].

To increase the protein content of the wheat buns, part of the wheat flour was substituted with milled TVP with a protein content above 50%. Since the protein content of the TVPs was substantially higher than the 14.6% in the wheat flour, the substitution resulted in buns with significantly increased protein contents. The extrusion process has been shown to increase protein digestibility [10], reduce off-flavours [17,18] and alter functionality [19,20]. Pea protein and faba bean protein have a high content of lysine, which is one of the limiting amino acids in cereals, but at the same time pea protein and faba bean protein are lower in the sulphur-containing amino acids methionine and cysteine [13,14]. In contrast, hemp protein and quinoa protein have a higher concentration of the sulphur-containing amino acids [7]. By combining these protein sources before extrusion, the aim is to gain a balanced amino acid composition in the bread. Protein quality is important for maintaining muscle mass, especially in older adults [8,9]. In this study, it was shown that the protein content of the buns increased by more than 100% when 50% of the flour was substituted with the TVPs.

However, this high substitution level had a considerable impact on both the baking quality and the sensory quality. The volume of the buns was significantly reduced, and it was found that the concentration of added TVP was negatively correlated to the final volume. The rising ability is dependent on the gluten content [15], and substituting the flour with TVPs reduces the gluten content, resulting in a decrease in volume. This has also been seen when substituting up to 15% of wheat flour with quinoa flour [21] and is therefore not specific to the TVPs. In contrast, another study showed that substituting 17.5% of the wheat flour with a combination of flour from different pulses did not change the specific volume [22], and therefore it might also be a question of the source of protein used in the substitution.

TVP3 contained 10% hemp, and the buns with TVP3 had a lower volume than the buns with TVP1 and TVP2 at 20% and 35%, whereas no difference was seen at 50%. The buns with TVP3 were also visibly different from the other buns, with another structure and a higher specific gravity, even though the hemp level was only 10% in the TVP3. This indicates a different functionality from that of TVP1 and TVP2. However, if as much as 50% of the wheat flour was substituted, the volume was low in all three types of TVP, and a limit for the addition could therefore be 35%. At this level, TVP1 and TVP2 were superior to TVP3 with respect to functionality. Besides the difference in functionality, TVP3 also gave a more intensive bitter taste than TVP1 and TVP2, further stressing the unsuitability of TVP3 at this level of addition.

The colour of the buns with TVP was darker than that of the reference bun, both of which were assessed by instrumental and sensory analysis for all three types of TVP. This is in accordance with another study in which part of the wheat flour was extruded, resulting in darker bread, especially in the crust but also in the crumb [23]. In our study, the colour of the crumb changed when analysed instrumentally (Table 3).

The sensory evaluation also revealed a darker crust in the buns with added TVPs (Figure 1). Brownness of the crust is formed by Maillard reactions between amino acids and carbohydrates [24]. In the aforementioned study on extruded wheat flour [23], the protein content was constant, and this might explain the lack of change in the crust colour. In our study, the darker crust in the buns with TVPs is most likely explained by an increased protein content [25]. The Maillard reaction responsible for the crust colour is also responsible for the bread flavour [26]. However, in our study, the darker crust was related to a change from a wheat flour flavour towards a rye flavour and was more than just an increase in the intensity of the flavour (Figure 1 and Figure 3).

In a set-up where food is delivered to elderly people, the buns need to be robust for storage and freezing/thawing. To evaluate the robustness of the buns, the sensory quality was compared on the day of baking (Day 0), the day after (Day 1) and after freezing and thawing on the table (FT) and in the microwave oven (FM). During storage and during freezing, staling occurs due to retrogradation of amylopectin [27]. In the buns with TVP, the content of amylopectin is lower, since the protein content is higher, and it could therefore be expected that the buns with TVP might be more robust against retrogradation. In particular, this could also be seen when comparing the buns thawed on the table and in the microwave oven, since the difference between these two treatments was minor in the TVP2 bun compared with the REF bun (Figure 3). Heating bread in a microwave oven is known to give a tougher crumb [28,29] and a lack of crust formation [28]. We also see that the crust was softer in the REF bun, while no difference was seen in the bun with TVP2 (Figure 4), indicating that the difference between the two types of buns was smaller in toughness at first bite after microwave thawing. Furthermore, in terms of storage time, the TVP2 bun was more robust, with the toughness of the crust at first bite in particular changing from day 0 to day 1 (Figure 3), and the change was larger for the REF bun than for the TVP2 bun (Figure 4). Taken together, it can be concluded that, even though the sensory quality of the fresh TVP2 bun was lower than that of the REF bun, the TVP2 bun was also more robust for storage, freezing and thawing.

Balancing the need for an increased protein content for nutritional reasons with an acceptable eating quality meant that we served the buns with 35% TVP to an expert panel to see if this level of addition was acceptable. No difference in liking was seen between the REF, TVP1 and TVP2, while the bitter and more compact TVP3 was given a lower score. Given information about the protein fortification and the protein quality, the expert panel were asked how likely it was that they would serve the buns to their customers. The likelihood of using the buns was lower for the REF bun than for the buns with added TVP, even though they liked it just as much as the other buns. Surprisingly, the likelihood of using the TVP3 bun was rated at the same level as the TVP1 and TVP2 buns, even though the sensory quality was lower, which shows how much emphasis the food professionals place on the protein intake of older adults. Overall, the study shows that, even though the sensory quality changed, an acceptable quality was achieved by the addition of 35% TVP, and it is therefore possible to significantly increase the protein content of the buns, thus contributing to a more nutritious breakfast for older adults.

## 5. Conclusions

By substituting wheat flour with texturized vegetable protein ingredients, it is possible to fortify bread and increase its protein content by 83% (up to 25% of DM) and still maintain an acceptable quality when evaluated by representatives of potential users. Combining different protein sources before extrusion showed that the combinations of pea and faba bean or pea, faba bean and quinoa were more suitable than the combination of pea, faba bean and hemp for this purpose. This shows that there is potential for producing high quality bread with superior protein content, for example for older adults who need a high level of protein intake in all their meals.

## Figures and Tables

**Table 1:** Recipe for buns with or without TVP.

Ingredient	Brand	REF (g)	TVP * 20% (g)	TVP * 35% (g)	TVP * 50% (g)
Dry yeast	Maltese cross yeast, The Danish Yeast Factories A/S	6	6	6	6
Sugar (refined)	Danish sugar, Nordic Sugar A/S	50	50	50	50
Aquafaba (chickpea)	Liquid from canned chickpeas (2.5 kg), Ponte Sisto APS	100	100	100	100
Table salt	Fine salt containing iodine, Azelis Denmark A/S	4	4	4	4
Vegan butter	Becel Gold-100% plant-based, Upfield Denmark A/S	80	80	80	80
Oat milk	Organic oat drink original, Naturli’ Foods A/S	240	260	280	320
Manitoba wheat flour	Molino Grassi Spa, Italy	530	424	344	265
TVP flour	Organic Plant Protein A/S	0	106	186	265
Total weight in dough		1010	1030	1050	1090

* TVP1, 2 and 3. TVP1: extrudate with 10% faba bean, 89% pea, 1% salt; TVP2: extrudate with 49% faba bean, 40% pea, 10% quinoa, 1% salt; TVP3: extrudate with 10% faba bean, 79% pea, 10% hemp, 1% salt. Twenty percent, 35% and 50% describe the amount of Manitoba flour that was substituted with TVP, based on weight.

**Table 2:** Sensory descriptors used in the descriptive analysis of the ten buns and the robustness evaluation.

	Descriptive Analysis	Robustness Evaluation
**Appearance**	Colour crust	Colour crust
	Uneven crust	Uneven crust
	Colour crumb	Colour crumb
	Airy crumb	Airy crumb
	Moist/doughy crumb	Doughy crumb
		Dry crumb
**Odour**	Yeast	Yeast
	Coarse flour (rye)	Rye bread
	Nutty	Walnut
	Sweet	Wheat bread
	Green (hemp)	
	Wet cardboard	
**Taste**	Sweet	Sweet
	Bitter	Bitter
	Salt	
**Flavour**	Yeast	Yeast
	Wheat flour	Wheat bread
	Coarse flour (rye)	Rye bread
	Nutty	Cardamom
	Fat	Hemp
		Burnt
**Consistency with fingers**	Elastic/spongy	Elastic/spongy
	Firm	Dry
		Moist
		Crumbly
**Consistency in mouth**	Crispy crust (1st bite)	Tough crust (1st bite)
	Crumbly	Crumbly
	Lumpy/sticky	Doughy
	Moist	
**Mouthfeel**	Astringent	Astringent
	Greasy	

**Table 3:** Average protein content, baking weight loss, rising ability (Vol) and colour measurements of all sample materials. Average protein content is based on three replicates, while all other measures are based on five replicates.

				Dough			Crust			Crumb		
Sample	Baking Weight Loss (%)	Vol (cm^3^)	Protein Content (%/DM)	L*	a*	b*	L*	a*	b*	L*	a*	b*
REF	10.0 ^a†^	163 ^a^	13.7 ^a^	84.6	1.9	14.4	45.1	17.0	26.1	77.5	2.7	15.2
TVP1-20	7.2 ^bc^	116 ^b^	20.1 ^b^	79.0	6.2	24.8	37.6	15.3	18.3	67.7	5.3	24.2
TVP1-35	8.4 ^abc^	86 ^cd^	25.1 ^c^	71.8	8.2	30.4	34.4	14.9	14.7	66.4	8.3	29.1
TVP1-50	9.2 ^ab^	61 ^e^	29.7 ^e^	67.4	10.6	32.3	41.6	18.7	25.0	61.5	9.6	30.4
TVP2-20	9.2 ^ab^	93 ^c^	20.5 ^b^	71.6	6.7	25.0	38.8	16.4	20.5	65.9	5.5	22.1
TVP2-35	9.2 ^ab^	76 ^d^	25.9 ^d^	69.0	7.3	26.2	36.2	17.9	18.7	61.1	6.8	24.3
TVP2-50	8.4 ^abc^	62 ^e^	30.2 ^e^	65.4	8.5	27.3	35.1	15.6	16.0	59.5	7.7	26.0
TVP3-20	6.8 ^c^	95 ^c^	20.6 ^b^	73.0	6.0	23.1	33.2	15.4	14.0	64.3	5.6	22.8
TVP3-35	7.2 ^bc^	69 ^e^	25.7 ^d^	64.3	7.9	26.4	46.2	16.9	29.3	58.6	7.3	25.6
TVP3-50	8.8 ^abc^	61 ^e^	30.2 ^e^	62.9	8.0	26.6	33.2	16.6	10.7	54.9	8.6	26.0
SE	0.5	2.6	0.1									

Colour is expressed in CIE colour space: L*; luminosity; a*; green to red; b*; blue to yellow. REF: reference sample with 0% added TVP; TVP1: extrudate with 10% faba bean, 89% pea, 1% salt; TVP2: extrudate with 49% faba bean, 40% pea, 10% quinoa, 1% salt; TVP3: extrudate with 10% faba bean, 79% pea, 10% hemp, 1% salt; 20: 20% addition; 35: 35% addition; 50: 50% addition. † Different letters indicate significant differences (*p* < 0.05) between samples.

**Table 4:** The samples’ average liking rating on a modified 9-point hedonic scale and standard error (SE). A paired *t*-test was used to test for significant effects of providing information.

	Liking No Info on Content		Likelihood of Use INFO on Content	
	Mean	SE	Mean	SE
REF	5.3 ^a†^	0.8	4.1 ^a^	1.0
TVP1-35	5.1 ^a^	0.5	5.7 ^a^	0.5
TVP2-35	5.2 ^a^	0.4	5.0 ^a^	0.6
TVP3-35	3.8 ^a^	0.6	5.0 ^a^	0.6

REF: Reference sample with 0% added TVP; TVP1: extrudate with 10% faba bean, 89% pea, 1% salt; TVP2: extrudate with 49% faba bean, 40% pea, 10% quinoa, 1% salt; TVP3: extrudate with 10% faba bean, 79% pea, 10% hemp, 1% salt; 35: 35% addition. † Different letters indicate significant differences (*p* < 0.05) between samples.

## Data Availability

The data presented in this study are available on request from the corresponding author. Although sensory and expert panel data have been anonymised, data are not publicly available.

## Supplementary Materials

The following supporting information can be downloaded at: https://www.mdpi.com/article/10.3390/foods11223647/s1, Table S1: Average rating from the sensory profiling of all samples measured on a 150 mm line scale. Standard error (SE) and significant *p*-values are valid for the TVP samples only (without REF). Table S2: Average rating of the samples from the sensory profile on robustness measured on a 150 mm line scale. Standard error (SE) and significant *p*-values are valid for all eight samples.
