# Supplementary material for: Texturized Vegetable Protein as a Source of Protein Fortification of Wheat Buns
Source: Foods. 2022 Nov 15;11(22):3647. doi: 10.3390/foods11223647 (PMC9689165; doi:10.3390/foods11223647)
Supplement: Supplementary file 1 [file foods-11-03647-s001.zip › foods-1974002-supplementary.pdf]

Supplemental

Table S1. Average rating from the sensory profiling of all samples measured on a 150 mm line scale. Standard error (SE) and significant p-values are valid for the TVP samples only (without REF).

		REF	TVP1			TVP2			TVP3			SE	Type	Conc.	Type*Conc.
**Sensory attributes**		**0**	**20**	**35**	**50**	**20**	**35**	**50**	**20**	**35**	**50**				
**Appearance**	Colour crust	24.2	88.8	80.5	120.3	90.8	91.4	105.9	62.8	112.5	91.4	5.9		0.000	0.000
	Uneven crust	13.2	26.7	74.3	107.0	59.7	59.3	95.0	36.0	97.8	72.4	10.7		0.000	0.000
	Colour crumb	10.4	46.0	65.2	85.2	60.0	74.7	92.3	57.0	97.7	104.8	5.8	0.000	0.000	0.031
	Airy crumb	115.2	90.8	45.2	21.9	65.8	38.7	28.3	75.9	26.4	28.2	4.6	0.026	0.000	0.002
	Moist/doughy crumb	35.8	39.7	81.0	96.3	61.0	69.2	95.1	49.1	94.0	92.8	10.1		0.000	
**Odour**	Sweet	93.2	55.8	62.3	53.4	64.3	58.2	49.9	73.2	53.6	39.7	8.3		0.001	0.054
	Yeast	60.3	42.4	47.3	40.8	44.3	44.2	39.4	46.1	31.4	44.8	5.0			0.015
	Coarse flour (rye)	9.3	55.9	75.9	82.4	59.0	81.7	80.7	64.3	82.3	86.8	7.9		0.000	
	Nutty	13.5	33.6	36.4	48.4	26.8	44.3	39.0	26.9	40.8	45.7	4.5		0.000	
	Green (hemp)	10.7	25.0	25.9	43.8	29.6	41.6	54.1	30.6	56.1	64.1	10.3	0.000	0.000	
	Wet cardboard	22.1	28.1	33.1	36.3	30.4	36.3	36.8	35.5	39.8	41.6	5.3			
**Taste**	Sweet	99.8	65.5	52.3	41.9	61.3	53.1	38.8	70.9	44.2	37.3	8.9		0.000	
	Salt	24.2	27.8	38.2	42.7	35.9	38.7	43.3	32.3	45.0	47.8	7.4		0.000	
	Bitter	12.8	31.5	46.4	71.1	29.6	48.4	74.9	32.8	62.6	80.6	6.3		0.000	
**Flavour**	Yeast	41.7	31.3	30.4	28.8	36.7	32.9	29.7	40.2	29.5	36.8	6.0			
	Nutty	13.6	31.2	35.9	53.0	29.9	38.0	38.3	30.3	45.3	36.7	5.4		0.001	0.044
	Wheat flour	116.0	69.1	43.9	20.4	67.8	36.8	20.3	65.2	30.4	26.5	5.6		0.000	
	Coarse flour (rye)	9.1	49.1	74.1	86.8	51.8	77.0	90.0	52.6	88.8	91.6	9.0		0.000	
	Fat	69.7	47.8	46.8	41.3	58.3	40.3	40.8	49.1	51.3	54.3	9.2			0.056
**Consistency with fingers**	Firmness	20.5	55.3	105.1	120.1	57.8	102.6	125.3	46.6	112.3	124.6	5.4		0.000	
	Elastic/spongy	109.1	81.2	24.7	26.3	72.6	40.5	19.4	73.3	28.4	20.0	5.7		0.000	
**Consistency in mouth**	Crispy crust (1st bite)	15.3	39.1	46.0	86.1	35.3	41.1	85.7	26.9	65.9	56.4	9.6		0.000	0.000
	Moist	104.4	62.8	40.8	28.6	63.9	45.0	25.0	76.4	39.7	35.3	6.3		0.000	
	Crumbling	24.3	45.8	86.3	112.1	47.4	79.7	106.8	53.8	82.4	105.3	9.4		0.000	
	Lumpy/sticky	105.3	71.8	69.8	30.1	92.4	63.9	35.2	76.5	54.1	50.6	7.9		0.000	0.026
**Mouthfeel**	Astringent	19.5	34.3	70.0	82.6	38.0	54.4	88.5	36.1	63.3	83.3	6.4		0.000	
	Greasy	71.7	42.5	41.6	43.1	53.5	44.9	48.8	47.9	45.1	43.7	8.4			

Table S2. Average rating of the samples from the sensory profile on robustness measured on a 150 mm line scale. Standard error (SE) and significant p-values are valid for all eight samples.

		REF				TVP2 35%				SE	Type	Treatment	Type* Treatment
**Sensory attributes**		**D0**	**D1**	**FT**	**M**	**D0**	**D1**	**FT**	**M**				
**Appearance**	Colour crust	36.7	50.8	38.7	44.6	109.0	126.2	132.9	119.4	8.9	0.000	0.082	
	Uneven crust	35.8	36.4	25.6	22.6	81.3	91.5	53.9	71.3	11.5	0.000	0.007	0.017
	Colour crumb	25.7	20.1	16.3	24.3	93.4	104.7	107.2	93.8	8.9	0.000		
	Airy crumb	117.8	117.1	130.8	115.3	50.8	55.2	48.8	44.8	8.4	0.000		
	Dry crumb	55.1	77.4	63.8	58.4	52.9	58.9	61.7	56.9	12.8			
	Doughy crumb	36.6	32.2	32.9	42.8	60.2	47.0	37.8	47.6	11.5	0.007	0.099	
**Odour**	Wheat	99.8	99.8	106.3	100.9	44.4	35.9	39.4	44.5	6.5	0.000		
	Rye bread	6.4	11.7	2.8	3.4	70.4	68.6	80.1	70.3	9.1	0.000		
	Yeast	35.8	37.8	35.7	40.2	33.0	22.3	25.5	28.3	13.5	0.001		
	Walnut	8.8	4.2	4.3	6.9	54.6	42.8	40.8	51.0	8.0	0.000	0.037	
**Taste**	Sweet	85.5	76.8	81.8	79.3	43.3	42.2	46.4	60.2	8.7	0.000		
	Bitter	4.6	13.3	3.4	5.4	43.5	49.0	53.6	37.4	6.01	0.000		
**Flavour**	Yeast	25.7	30.4	25.5	37.3	25.7	18.0	18.8	24.1	9.3	0.030		
	Wheat	95.0	81.2	88.7	89.9	37.5	33.4	27.7	49.5	7.7	0.000		
	Burned	0.3	12.5	1.7	2.3	69.4	56.3	81.4	58.9	8.2	0.000		0.054
	Rye bread	0.4	16.1	0.0	2.0	59.4	57.9	68.3	46.9	9.6	0.000		
	Cardamom	27.3	17.4	20.0	20.2	17.0	15.3	11.6	16.5	7.7	0.013		
	Hemp	0.0	0.0	0.0	0.0	19.1	19.0	20.8	17.5	7.7	0.000		
**Consistency with fingers**	Elastic/spongy	100.3	89.8	106.0	101.3	36.4	24.8	43.8	49.5	8.5	0.000	0.020	
	Dryness	75.8	66.9	80.1	77.5	96.3	80.3	84.1	97.8	12.2	0.083		
	Moist	47.9	43.3	49.0	58.4	35.2	25.8	44.7	51.7	9.0	0.077		
	Crumbling	35.9	50.2	33.5	40.3	81.9	102.6	87.0	80.8	11.7	0.000	0.046	
**Consistency in mouth**	Tough crust (1st bite)	101.2	61.9	121.2	94.2	45.7	29.8	63.8	68.3	9.9	0.000	0.000	
	Doughy	55.7	57.7	49.3	56.3	46.9	45.8	48.0	37.3	11.5			
	Crumbling	16.8	42.6	12.7	21.8	80.1	95.3	82.1	77.3	11.9	0.000		
**Mouthfeel**	Astringent	9.5	15.8	12.5	10.5	43.3	51.4	51.0	43.5	9.9	0.000		
